# Academic performance in children of mothers with perinatal depressive disorder

**DOI:** 10.1177/00048674261457299

**Published:** 2026-06-23

**Authors:** Biruk Shalmeno Tusa, Rosa Alati, Getinet Ayano, Berihun Dachew

**Affiliations:** 1 School of Population Health, Curtin University, Perth, WA, Australia; 2 Department of Epidemiology and Biostatistics, College of Health and Medical Sciences, Haramaya University, Haramaya, Ethiopia; 3 School of Public Health, The University of Queensland, Brisbane, QLD, Australia

**Keywords:** Maternal perinatal depression, NAPLAN, children’s academic performance

## Abstract

**Background:**

Maternal perinatal depression is a well-established risk factor for adverse child developmental outcomes; however, its long-term association with academic achievement remains unclear. This study examined its association with children’s academic performance across multiple stages of schooling.

**Methods:**

Data were drawn from the New South Wales Perinatal Data Collection, linked with the New South Wales Admitted Patient Data Collection and the New South Wales National Assessment Program – Literacy and Numeracy. Maternal perinatal depressive disorders were identified from hospital admission records using the International Classification of Diseases, 10th Revision, Australian Modification codes. Academic performance was assessed using the National Assessment Program – Literacy and Numeracy results in Grades 3, 5 and 7. Outcomes were classified as meeting or not meeting the national minimum standards in reading, spelling, writing, grammar and numeracy. Generalised estimating equations and propensity score matching were applied to examine associations.

**Results:**

After adjustment and matching, maternal perinatal depressive disorders were associated with increased odds of children not meeting the national minimum standards in reading (odds ratio = 1.24, 95% confidence interval = [1.10, 1.41]), spelling (odds ratio = 1.28, 95% confidence interval = [1.13, 1.46]), writing (odds ratio = 1.32, 95% confidence interval = [1.19, 1.47]) and grammar (odds ratio = 1.18, 95% confidence interval = [1.06, 1.33]), but not numeracy (odds ratio = 1.10, 95% confidence interval = [0.96, 1.27]). Associations were more consistent for antenatal depression, while postnatal depression was associated with reading and writing only.

**Conclusions:**

Children of mothers with perinatal depressive disorders showed an association with suboptimal academic performance, particularly in literacy-related domains. The stronger associations observed for antenatal depression suggest that early gestational exposure may affect foundational cognitive and socio-emotional development, highlighting the importance of timely identification and treatment of maternal depression during pregnancy to support long-term educational outcomes.

## Background

Depression is a major contributor to the global disease burden Vos et al., (2020). Maternal perinatal depression, which includes both antenatal (during pregnancy) and postnatal (within the first year after birth) periods (Gavin et al., 2005), is characterised by persistent sadness, heightened anxiety and fatigue that may interfere with daily functioning, including self-care and the capacity to care for an infant (Lund and Town, 2016). Globally, an estimated 21% of women experience antenatal depression (Yin et al., 2021) and 14% experience postnatal depression (Liu et al., 2022). In Australia, prevalence estimates range from 10% to 15% for antenatal depression and 9–14% for postnatal depression (Foundation, 2022; Wang et al., 2021).

Maternal perinatal depressive disorder is associated with a wide range of adverse health outcomes in offspring, including impairments in cognition, neuromotor skills, behavioural regulation, social-emotional and physical development, as well as adverse birth outcomes (Bluett-Duncan et al., 2021; Dadi et al., 2020; Porter et al., 2019; Rogers et al., 2020; Stein et al., 2014; Van den Bergh et al., 2020). These effects may operate through multiple developmental pathways. For antenatal depression, maternal stress and hypothalamic–pituitary–adrenal (HPA) axis dysregulation can alter the intrauterine environment, affecting foetal brain development and later cognitive functioning (Cattarinussi et al., 2021; Nemoda and Szyf, 2017; Robinson et al., 2019; Severo et al., 2023). Postnatal depression may affect offspring via environmental and parenting mechanisms, including reduced cognitive stimulation, delayed language exposure and compromised mother–infant interactions, which are critical for early learning and socio-emotional development (McLeod et al., 2012; Sasayama et al., 2025; Şipoş et al., 2025).

Several studies have also examined the relationship between maternal depression and academic outcomes in offspring, although the findings remain inconsistent (Ahun et al., 2022; Augustine and Crosnoe, 2010; Ayano et al., 2022; Bechtiger et al., 2022; Claessens et al., 2015; Dahlen, 2016; Lin et al., 2017; Murray et al., 2010; Pearson et al., 2016; Psychogiou et al., 2020; Shen et al., 2016). For instance, a Canadian cohort study by Ahun et al. (2022) reported an association between maternal depressive symptoms and lower mathematics exam scores in children. In contrast, an Australian cohort study by Ayano et al. (2022) did not observe such an association. The existing evidence on the link between maternal depression and academic outcomes in children also exhibits several methodological limitations. Some used non-diagnostic screening tools to assess maternal depression (Ahun et al., 2022; Augustine and Crosnoe, 2010; Ayano et al., 2022; Bechtiger et al., 2022; Claessens et al., 2015; Dahlen, 2016; Murray et al., 2010; Pearson et al., 2016; Psychogiou et al., 2020), while others were based on small sample sizes, limiting statistical power and generalisability (Ahun et al., 2022; Augustine and Crosnoe, 2010; Ayano et al., 2022; Bechtiger et al., 2022; Murray et al., 2010). Furthermore, important potential confounders, such as socioeconomic disadvantage (Murray et al., 2010), obstetric complications (Ahun et al., 2022; Augustine and Crosnoe, 2010; Ayano et al., 2022; Bechtiger et al., 2022; Claessens et al., 2015; Dahlen, 2016; Murray et al., 2010; Pearson et al., 2016; Psychogiou et al., 2020; Shen et al., 2016), adverse birth outcomes (Ahun et al., 2022; Augustine and Crosnoe, 2010; Bechtiger et al., 2022; Claessens et al., 2015; Murray et al., 2010; Psychogiou et al., 2020; Shen et al., 2016), and maternal psychiatric and substance use disorders (Ahun et al., 2022; Augustine and Crosnoe, 2010; Bechtiger et al., 2022; Claessens et al., 2015; Dahlen, 2016; Murray et al., 2010; Pearson et al., 2016; Psychogiou et al., 2020; Shen et al., 2016) have not been consistently accounted for. In addition, academic performance has often been measured at a single point in time, despite its variation across different stages of schooling (Ahun et al., 2022; Ayano et al., 2022; Bechtiger et al., 2022; Murray et al., 2010; Ng-Knight et al., 2018; Pearson et al., 2016; Shen et al., 2016).

To address these gaps, this study examined the association between maternal perinatal depressive disorder and offspring academic performance using a large population-based dataset from New South Wales (NSW), Australia. Academic outcomes were assessed at three time points over a 5-year period to capture variation over time. To control for confounding, we first adjusted for a broad range of maternal, perinatal and sociodemographic factors. We then applied propensity score matching (PSM) to balance the measured baseline characteristics between the exposed and unexposed groups. The findings contribute population-level evidence on the association between perinatal depressive disorders and later academic performance and may inform strategies for monitoring and supporting children potentially at risk.

## Methods

### Data sources

Data were sourced from the New South Wales (NSW) Perinatal Data Collection (PDC) and linked to the NSW Admitted Patient Data Collection (APDC) for both mothers and children, as well as the NSW National Assessment Program – Literacy and Numeracy (NAPLAN) dataset. The study cohort comprised all children born in NSW between 2003 and 2005 who later participated in NAPLAN assessments in Grades 3, 5 and 7. The final analytic sample consisted of 206,452 children assessed at Grade 3, 206,068 at Grade 5 and 200,179 at Grade 7, resulting in a total of 612,699 assessment time points.

### Outcomes variables

Children’s academic performance was assessed using NAPLAN data collected in Grades 3, 5 and 7 (8–14 years). NAPLAN is an annual, standardised assessment administered across all government and non-government schools in Australia. It evaluates five domains: four literacy domains – reading, spelling, grammar, writing, and one numeracy domain. Children scoring below the national minimum standard (NMS) in any domain were classified as not meeting the NMS.

### Exposure variables

Maternal perinatal depressive disorder was identified using diagnostic codes F32–F39 from the International Classification of Diseases, 10th Revision, Australian Modification (ICD-10-AM). These diagnoses were sourced from the APDC and included both principal and secondary diagnoses. Antenatal depressive disorder refers to depressive episodes diagnosed between conception and delivery, while postnatal depressive disorder refers to episodes diagnosed from delivery up to 52 weeks postpartum.

### Potential confounders

Potential confounding variables were obtained from the PDC, APDC and NAPLAN datasets. These included maternal age, socio-economic indicators, language background other than English, child sex, birth order, plurality of birth, parity, presentation, onset of labour, mode of delivery, preterm birth, low birth weight, low Apgar score, neonatal intensive care unit admission, special care nursery admission, maternal antenatal infection, smoking, anaemia, chronic hypertension, pregnancy-induced hypertension, pre-existing diabetes, gestational diabetes, maternal diagnoses of perinatal anxiety disorder, bipolar disorder, schizophrenia, alcohol use disorder and substance use disorder.

### Statistical analysis

Children’s academic performance was summarised across key maternal and child characteristics. To examine the association between maternal perinatal depressive disorder and children’s academic outcomes over time, generalised estimating equation (GEE) models were employed with a binomial distribution, log link function and unstructured correlation structure. Results were reported as odds ratios (ORs) with 95% confidence intervals (CIs). As part of the sensitivity analyses, associations were also assessed using a complete-case sample of 184,768 children with data available at all three assessment points. In addition, separate logistic regression models were fitted at each grade level (i.e. Grades 3, 5 and 7) to evaluate grade-specific associations.

To improve comparability between exposed and non-exposed groups, PSM was applied using nearest neighbour matching with a 4 to 1 ratio and a calliper width of 0.25. The propensity score was estimated using a generalised linear model with a binomial distribution and included all predefined confounders listed above. For antenatal depression exposure, matching was performed on all predefined covariates except labour onset, mode of delivery, presentation, preterm birth, low birth weight, low Apgar score, admission to the neonatal intensive care unit and admission to a special care nursery, as these variables may lie on the causal pathway between antenatal depression and subsequent academic outcomes. For analyses of perinatal or postnatal depressive disorder, these perinatal factors were included in the matching procedure where temporally appropriate. Matching was conducted without replacement using the MatchIt package in R (Stuart et al., 2011). Balance was assessed using standardised mean differences and propensity score distribution plots (Supplemental Figures S1–S6). Adequate balance was defined as post-matching standardised mean differences below 0.1 and overlapping propensity score distributions with closely aligned peaks (Stuart et al., 2011). The matched dataset was then analysed using the same GEE and logistic regression model specifications as applied in the primary analyses.

## Results

### Characteristics of study participants

**Figure 1.**
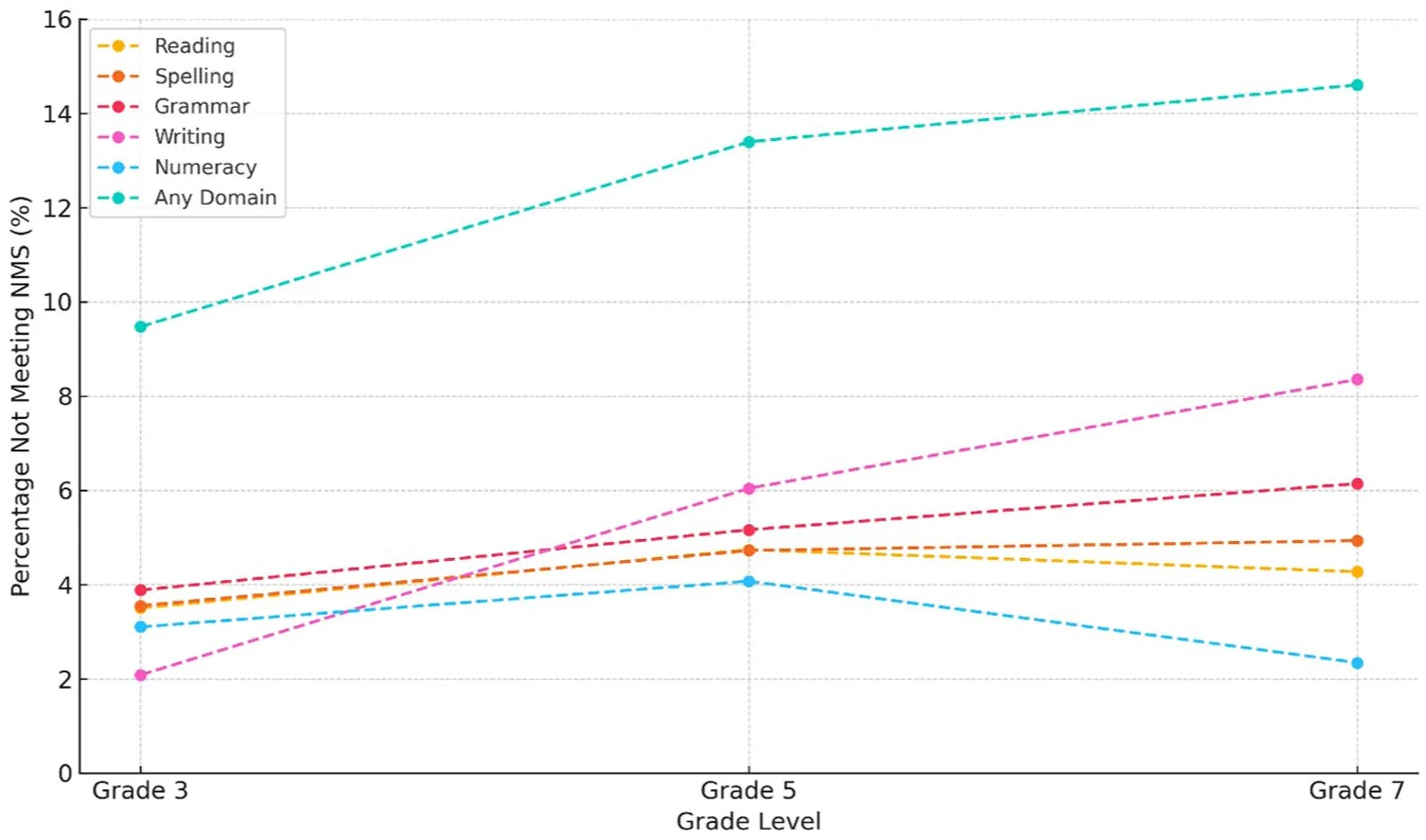
Proportion of children not meeting National Minimum Standards (NMS).

Of the 206,452 mother-offspring pairs included in the baseline, 1712 mothers (0.8%) were diagnosed with antenatal depressive disorder, 881 (0.4%) with postnatal depressive disorder and 2471 (1.2%) with perinatal depressive disorder. Regarding NAPLAN academic performance, most children met the NMS across all domains in Grades 3, 5 and 7. However, the proportion of students not meeting the standards gradually increased over time, particularly in the literacy domains. In reading and spelling, the percentage of students below the NMS rose from approximately 3.5% in Grade 3 to around 4.3–4.9% in Grade 7. Grammar followed a similar upward trend, with the proportion below the standard increasing from about 3.9% in Grade 3 to 6.1% in Grade 7. Writing showed the most substantial change, with only 2.1% of students not meeting the NMS in Grade 3, rising to 8.4% by Grade 7. When performance across any domain was considered, the proportion of students not meeting the NMS rose from 9.5% in Grade 3 to 14.6% in Grade 7 (Figure 1).

Table 1 shows the characteristics of children and their mothers, stratified by educational performance. Children were less likely to meet NMS if they were born to younger or nulliparous mothers, delivered via assisted or caesarean birth, or exposed in utero to maternal factors including chronic hypertension, pre-existing diabetes, anaemia, infections, mental health disorders, substance use or smoking. Poorer NMS outcomes were also observed among male children, those born preterm or with low birthweight, part of a multiple birth, with low Apgar scores, admitted to neonatal intensive care, or from non-English-speaking backgrounds.

**Table 1.** Maternal and offspring characteristics by outcome status.

Characteristics	Any domain in year 3		Any domain in year 5		Any domain in year 7	
	NMS not met *n* = 19,557 (%)	NMS met *n* = 186,685 (%)	NMS not met *n* = 27,593 (%)	NMS met *n* = 178,475(%)	NMS not met *n* = 29,219 (%)	NMS met *n* = 170,960 (%)
**Maternal characteristics**						
Maternal age						
12–19	2514 (12.85)	9083 (4.86)	3334 (12.08)	8240 (4.62)	3430 (11.74)	7346 (4.30)
20–24	3894 (19.91)	20,738 (11.10)	5400 (19.57)	19,059 (10.68)	5648 (19.33)	17,674 (10.34)
25–29	5405 (27.64)	51,633 (27.63)	7689 (27.87)	49,040 (27.48)	8279 (28.33)	46,887 (27.43)
30–34	4972 (25.42)	66,194 (35.42)	7195 (26.08)	63,928 (35.82)	7641 (26.15)	61,965 (36.25)
⩾35	2772 (14.17)	39,247 (21.4)	3975 (14.41)	38,208 (21.41)	4221 (14.45)	37,088 (21.69)
Socio-economic indexes for area						
First most disadvantage	7937 (40.58)	44,088 (23.59)	11,403 (41.33)	41,909 (23.48)	10,509 (42.9)	37,521 (23.4)
Second most disadvantage	5244 (26.81)	37,656 (20.15)	7424 (26.91)	37,571 (21.05)	5851 (23.9)	34,185 (21.3)
Third lowest disadvantage	3957 (20.23)	45,280 (24.23)	5487 (19.89)	44,116 (24.72)	5429 (22.1)	42,416 (26.5)
Fourth lowest disadvantage	2419 (12.37)	59,871 (32.03)	3279 (11.88)	54,879 (30.75)	2721 (11.1)	46,070 (28.8)
Parity						
Nulliparity	6216 (31.78)	79,516 (42.55)	9203 (33.35)	76,282 (42.74)	9993 (34.20)	73,597 (43.05)
Low multiparity (1–3)	11,679 (59.72)	102,347 (54.76)	16,246 (58.88)	97,664 (54.72)	17,161 (58.73)	93,237 (54.54)
Grand multipara (>3)	1662 (8.50)	5032 (2.69)	2144 (7.77)	4529 (2.54)	2065 (7.07)	4126 (2.41)
Presentations						
Vertex	18,494 (94.56)	177,105 (94.76)	26,131 (94.70)	169,086 (94.74)	27,678 (94.73)	161,908 (94.71)
Breech	890 (4.55)	8396 (4.49)	1236 (4.48)	8043 (4.51)	1286 (4.40)	7775 (4.55)
Other^a^	173 (0.88)	1394 (0.88)	226 (0.82)	1346 (0.75)	255 (0.87)	1277 (0.75)
Labour onset						
Spontaneous	12,220 (62.48)	110,657 (59.21)	17,014 (61.66)	105,596 (59.17)	17,842 (61.06)	101,296 (59.25)
Induced	4584 (23.44)	46,093 (24.66)	6679 (24.21)	43,872 (24.58)	7119 (24.36)	41,860 (24.49)
No labour	2753 (14.08)	30,134 (16.12)	3900 (14.13)	28,997 (16.25)	4257 (14.57)	27,795 (16.26)
Mode of delivery						
Spontaneous vaginal	13,403 (68.53)	114,426 (61.22)	18,651 (67.59)	108,813 (60.97)	19,599 (67.08)	103,870 (60.76)
Assisted^b^	1336 (6.83)	20,154 (10.78)	2000 (7.25)	19,419 (10.88)	2194 (7.51)	18,786 (10.99)
Caesarean section	4818 (24.64)	52,303 (27.99)	6942 (25.16)	50,233 (28.15)	7426 (25.41)	48,292 (28.25)
Chronic hypertension						
Yes	217 (1.11)	1899 (1.02)	330 (1.20)	1790 (1.00)	374 (1.28)	1681 (0.98)
No	19,340 (98.89)	184,996 (98.98)	27,263 (98.80)	176,685 (99.00)	28,845 (98.7)	169,279 (99.02)
Pregnancy-induced hypertension						
Yes	1725 (8.82)	15,006 (8.03)	2560 (9.28)	14,176 (7.94)	2766 (9.47)	13,456 (7.87)
No	17,832 (91.18)	171,889 (91.97)	25,033 (90.72)	164,299 (92.06)	26,453 (90.53)	157,504 (92.13)
Pre-existing diabetes						
Yes	146 (0.75)	987 (0.53)	189 (0.68)	925 (0.52)	194 (0.66)	890 (0.52)
No	19,411 (99.25)	185,908 (99.47)	27,404 (99.32)	177,550 (99.48)	29,025 (99.34)	170,070 (99.48)
Gestational diabetes						
Yes	1006 (5.14)	9193 (4.92)	1431 (5.19)	8755 (4.91)	1490 (5.10)	8472 (4.96)
No	18,551 (94.86)	177,702 (95.08)	26,162 (94.81)	169,720 (95.09)	27,729 (94.90)	162,488 (95.04)
Antenatal anaemia						
Yes	589 (3.01)	3485 (1.86)	759 (2.75)	3318 (1.86)	763 (2.61)	3161 (1.85)
No	18,968 (96.99)	183,410 (98.14)	26,834 (97.25)	175,157 (98.14)	28,456 (97.39)	167,799 (98.15)
Antenatal infection						
Yes	228 (1.17)	1362 (0.73)	229 (1.08)	1271 (0.71)	310 (1.06)	1177 (0.69)
No	19,329 (98.83)	185,533 (99.27)	27,294 (98.92)	177,204 (99.29)	28,909 (98.94)	169,783 (99.31)
Perinatal depressive disorder						
Yes	406 (2.08)	2065 (1.10)	543 (1.97)	1932 (1.08)	584 (2.00)	1768 (1.03)
No	19,151 (97.92)	184,830 (98.90)	27,050 (98.03)	176,543 (98.92)	28,635 (98.00)	169,192 (99.0)
Antenatal depressive disorder						
Yes	289 (1.48)	1423 (0.76)	399 (1.45)	1314 (0.74)	426 (1.46)	1206 (0.71)
No	19,268 (98.52)	185,472 (99.24)	27,194 (98.55)	177,161 (99.26)	28,793 (98.57)	169,754 (99.29)
Postnatal depressive disorder						
Yes	140 (0.72)	741 (0.40)	179 (0.65)	700 (0.39)	195 (0.67)	636 (0.4)
No	19,417 (99.28)	186,154 (99.60)	27,414 (99.35)	177,775 (99.61)	29,024 (99.33)	170,324 (99.6)
Perinatal anxiety disorder						
Yes	338 (1.73)	2867 (1.53)	470 (1.70)	2741 (1.54)	514 (1.76)	2526 (1.48)
No	19,219 (98.27)	184,028 (98.47)	27,123 (98.30)	175,734 (98.46)	28,705 (98.24)	168,434 (98.52)
Perinatal bipolar disorder						
Yes	35 (0.18)	229 (0.12)	61 (0.22)	212 (0.12)	54 (0.18)	193 (0.11)
No	19,522 (99.82)	186,666 (99.88)	27,532 (99.78)	178,263 (99.88)	29,165 (99.82)	170,767 (99.89)
Perinatal schizophrenia disorders						
Yes	73 (0.37)	240 (0.13)	87 (0.32)	222 (0.12)	91 (0.31)	192 (0.11)
No	19,484 (99.63)	186,655 (99.87)	27,506 (99.68)	178,253 (99.88)	29,128 (99.69)	170,768 (99.89)
Perinatal alcohol use disorder						
Yes	121 (0.62)	267 (0.14)	154 (0.56)	236 (0.13)	147 (0.50)	205 (0.12)
No	19,436 (99.38)	186,628 (99.86)	27,439 (99.44)	178,239 (99.87)	29,072 (99.50)	170,755 (99.88)
Perinatal substance use disorder^c^						
Yes	669 (3.42)	1637 (0.88)	887 (3.21)	1384 (0.78)	865 (2.96)	1166 (0.68)
No	18,888 (96.58)	185,258 (99.12)	26,706 (96.79)	177,091 (99.22)	28,354 (97.04)	169,794 (99.32)
Antenatal smoking						
Yes	6108 (31.23)	22,311 (12.00)	8205 (29.74)	20,055 (11.30)	8221 (28.14)	17,920 (10.55)
No	13,441 (68.73)	164,474 (88.00)	19,376 (70.26)	158,316 (88.70)	20,989 (71.86)	152,932 (89.45)
**Offspring characteristics**						
Sex of the baby						
Male	12,556 (64.20)	92,868 (49.69)	17,656 (63.99)	87,596 (49.08)	19,457 (66.59)	82,463 (48.24)
Female	6997 (36.80)	93,906 (50.31)	9931 (36.01)	90,759 (51.92)	9753 (33.41)	88,381 (51.76)
Plurality of birth						
Singleton	18,871 (96.49)	181,543 (97.14)	26,680 (96.69)	173,340 (97.12)	28,268 (96.75)	165,982 (97.09)
Twins and above	686 (3.51)	5352 (2.86)	913 (3.31)	5135 (2.88)	951 (3.25)	4978 (2.91)
Birth order						
First	19,210 (98.23)	184,219 (98.57)	27,119 (98.28)	175,907 (98.56)	28,754 (98.41)	168,445 (98.53)
Second and above	347 (1.77)	2676 (1.43)	474 (1.72)	2568 (1.44)	465 (1.59)	2515 (1.47)
Preterm birth						
Yes	1678 (8.58)	10,527 (5.63)	2278 (8.26)	9994 (5.60)	2334 (7.99)	9454 (5.53)
No	17,879 (91.42)	176,368 (94.37)	25,315 (91.74)	168,481 (94.40)	26,885 (92.01)	161,506 (94.47)
Low birth weight						
Yes	1600 (8.18)	8815 (4.72)	2184 (7.92)	8303 (4.65)	2183 (7.47)	7839 (4.59)
No	17,957 (91.82)	178,080 (95.28)	25,409 (92.08)	170,172 (95.35)	27,036 (92.53)	163,121 (95.41)
Low Apgar score						
Yes	314 (1.61)	1920 (1.03)	413 (1.50)	1809 (1.01)	421 (1.44)	1715 (1.00)
No	19,243 (98.39)	184,975 (98.97)	27,180 (98.50)	176,666 (98.99)	28,798 (98.56)	169,245 (99.00)
Admitted to neonatal intensive care unit						
Yes	710 (3.64)	4000 (2.15)	972 (3.52)	3794 (2.14)	989 (3.38)	3576 (2.09)
No	18,846 (96.36)	182,872 (97.85)	26,619 (96.48)	174,659 (97.86)	28,227 (96.60)	167,364 (97.90)
Admitted to a special care nursery						
Yes	3394 (17.35)	25,504 (13.65)	4775 (17.31)	24,044 (13.47)	4978 (17.04)	22,905 (13.40)
No	16,160 (82.63)	161,303 (86.31)	22,813 (82.68)	154,336 (86.47)	24,237 (82.95)	147,961 (86.55)
Language background other than English						
Yes	4380 (22.40)	52,316 (27.99)	6349 (23.01)	51,019 (28.59)	6171 (21.12)	46,857 (27.41)
No	15,064 (77.03)	132,398 (70.84)	21,060 (76.32)	125,475 (70.30)	22,721 (77.76)	120,864 (70.70)
Not stated	113 (0.58)	2181 (1.17)	184 (0.67)	1981 (1.11)	327 (1.12)	3239 (1.89)

NMS: National Minimum Standards.

a Other includes face, brow and shoulder/transverse.

b Assisted includes forceps and vacuum.

c Substance use disorders include alcohol, tobacco, cannabis, opioids, cocaine, hallucinogens and stimulants.

NB: Percentages represent column percentages.

### Associations between maternal perinatal depressive disorders and children’s academic performance

Table 2 presents the associations between maternal perinatal depressive disorders and children’s academic performance. In the adjusted model, maternal perinatal depressive disorders were associated with increased odds of not meeting the NMS in reading, spelling, grammar and writing. Similar patterns were observed in the matched models: reading (OR = 1.24, 95% CI = [1.10, 1.41]), spelling (OR = 1.28, 95% CI = [1.13, 1.46]), grammar (OR = 1.18, 95% CI = [1.06, 1.33]) and writing (OR = 1.32, 95% CI = [1.19, 1.47]). Antenatal depressive disorders were consistently associated with higher odds of not meeting the NMS across all literacy domains following matching: reading (OR = 1.25, 95% CI = [1.08, 1.44]), spelling (OR = 1.43, 95% CI = [1.23, 1.66]), grammar (OR = 1.22, 95% CI = [1.06, 1.39]) and writing (OR = 1.39, 95% CI = [1.23, 1.58]). In the unmatched adjusted analysis, postnatal depressive disorders were associated with increased odds of children not meeting the NMS in reading, spelling, writing and numeracy. After matching, associations remained evident only for reading (OR = 1.25; 95% CI = [1.01, 1.54]) and writing (OR = 1.29; 95% CI = [1.08, 1.54]). No associations were observed for numeracy in any exposure group within the matched models. Sensitivity analyses using the complete-case sample (Supplemental Table S1) and logistic regression models at each time point (Supplemental Table S2) revealed comparable results.

**Table 2.** Bivariable and multivariable analysis for the association between maternal depressive disorder and educational outcomes in children.

Outcome variables	Antenatal depressive disorder				Postnatal depressive disorder				Perinatal depressive disorder			
	Unadjusted OR [95% CI]	*p*-value	Adjusted OR^a^ [95% CI]	*p*-value	Unadjusted OR [95% CI]	*p*-value	Adjusted OR^a,b^ [95% CI]	*p*-value	Unadjusted OR [95% CI]	*p*-value	Adjusted OR^a^ [95% CI]	*p*-value
**Reading domain**												
Unmatched	1.91 [1.69, 2.16]	<0.01	1.25 [1.09, 1.43]	<0.01	1.93 [1.62, 2.31]	<0.01	1.22 [1.00, 1.49]	0.05	1.89 [1.70, 2.10]	<0.01	1.24 [1.11, 1.40]	<0.01
Matched	1.32 [1.15, 1.52]^c^	<0.01	1.25 [1.08, 1.44]^c^	<0.01	1.29 [1.06, 1.58]^d^	0.01	1.25 [1.01, 1.54]^d^	0.04	1.30 [1.16, 1.46]^d^	<0.01	1.24 [1.10, 1.41]^d^	<0.01
**Spelling domain**												
Unmatched	2.12 [1.86, 2.41]	<0.01	1.36 [1.18, 1.56]	<0.01	2.06 [1.71, 2.48]	<0.01	1.27 [1.03, 1.55]	0.02	2.03 [1.81, 2.26]	<0.01	1.31 [1.15, 1.48]	<0.01
Matched	1.52 [1.32, 1.75]^c^	<0.01	1.43 [1.23, 1.66]^c^	<0.01	1.27 [1.04, 1.56]^d^	0.02	1.20 [0.97, 1.49]^d^	0.10	1.36 [1.21, 1.54]^d^	<0.01	1.28 [1.13, 1.46]^d^	<0.01
**Grammar domain**												
Unmatched	1.84 [1.63, 2.07]	<0.01	1.22 [1.07, 1.38]	<0.01	1.69 [1.41, 2.01]	<0.01	1.09 [0.91, 1.32]	0.35	1.77 [1.60, 1.96]	<0.01	1.19 [1.07, 1.32]	<0.01
Matched	1.30 [1.14, 1.47]^c^	<0.01	1.22 [1.06, 1.39]^c^	<0.01	1.18 [0.97, 1.43]^d^	0.10	1.13 [0.92, 1.38]^d^	0.26	1.24 [1.11, 1.38]^d^	0.01	1.18 [1.06, 1.33]^d^	<0.01
**Writing domain**												
Unmatched	2.17 [195, 2.42]	<0.01	1.39 [1.23, 1.56]	<0.01	2.06 [1.77, 2.40]	<0.01	1.23 [1.04, 1.46]	0.02	2.10 [1.92, 2.30]	<0.01	1.19 [1.07, 1.32]	<0.01
Matched	1.44 [1.28, 1.63]^c^	<0.01	1.39 [1.23, 1.58]^c^	<0.01	1.30 [1.09, 1.54]^d^	<0.01	1.29 [1.08, 1.54]^d^	0.01	1.34 [1.21, 1.49]^d^	<0.01	1.32 [1.19, 1.47]^d^	<0.01
**Numeracy domain**												
Unmatched	1.67 [1.45, 1.94]	<0.01	1.09 [0.92, 1.26]	0.34	1.90 [1.55, 2.34]	<0.01	1.25 [1.00, 1.56]	0.05	1.68 [1.44, 1.96]	<0.01	1.11 [0.97, 1.27]	0.13
Matched	1.11 [0.95, 1.31]^c^	0.18	1.04 [0.89, 1.23]^c^	0.60	1.25 [1.00, 1.57]^d^	0.05	1.21 [0.95, 1.53]^d^	0.11	1.16 [1.01, 1.33]^d^	0.04	1.10 [0.96, 1.27]^d^	0.16
**Any domain**												
Unmatched	2.01 [1.83, 2.19]	<0.01	1.36 [1.23, 1.50]	<0.01	1.75 [1.54, 2.00]	<0.01	1.12 [0.97, 1.30]	0.14	1.89 [1.75, 2.04]	<0.01	1.29 [1.18, 1.40]	<0.01
Matched	1.42 [1.29, 1.57]^c^	<0.01	1.35 [1.21, 1.50]^c^	<0.01	1.22 [1.06, 1.41]^d^	0.01	1.15 [0.98, 1.34]^d^	0.08	1.34 [1.23, 1.46]^d^	<0.01	1.28 [1.17, 1.40]^d^	<0.01

a Models adjusted for maternal age, socio-economic indicators, language background other than English, sex of the baby, birth order, plurality of birth parity, presentation, labour onset, mode of delivery, preterm, low birth weight, low Apgar score, admitted to neonatal intensive care unit, admitted to a special care nursery, maternal antenatal infection, maternal antenatal smoking, maternal antenatal anaemia, chronic hypertension, pregnancy-induced hypertension, pre-existing diabetes, gestational diabetes, perinatal anxiety disorder, perinatal bipolar disorder, perinatal schizophrenia disorder, perinatal alcohol use disorder and perinatal substance use disorder.

b Further adjusted for antenatal depressive disorder.

c The sample matched for all variables in the models except labour onset, mode of delivery, presentation, preterm, low birth weight, low Apgar score, admitted to the neonatal intensive care unit and admitted to a special care nursery.

d The sample matched for all variables in models.

## Discussion

### Main findings

In this population-based cohort study, we examined the association between maternal perinatal depressive disorders and offspring academic performance across multiple stages of schooling. After accounting for a range of maternal, perinatal and sociodemographic factors and applying PSM, we found that exposure to maternal perinatal depressive disorders was consistently associated with increased odds of children not meeting the NMS in literacy domains – reading, spelling, writing and grammar, but not in numeracy. Associations were stronger and more consistent for antenatal depression compared to postnatal depression.

A population-based cohort study from Sweden has examined the association between maternal antenatal depressive disorder and academic performance at age 16 (Shen et al., 2016). Consistent with our findings, this study reported a significant association between maternal antenatal depressive disorder and poorer school performance. The strength and rigour of our study, including a large sample size that ensured adequate statistical power, repeated measurements of educational outcomes across multiple stages of schooling, and the use of PSM to reduce confounding, enhance the robustness of the observed associations. These findings highlight the potential impact of maternal mental health during pregnancy on children’s long-term cognitive and educational development.

Our finding of an association between postnatal depressive disorder and poorer performance in reading and writing is also aligned with earlier research (Ahun et al., 2022; Claessens et al., 2015; Dahlen, 2016; Pearson et al., 2016). For example, a large cohort study in the United States identified a significant negative effect of postnatal depression on children’s reading scores by third grade (Dahlen, 2016). Similarly, a Canadian cohort study reported a negative association between maternal postnatal depressive symptoms and children’s reading and writing performance (Ahun et al., 2022). In contrast, an Australian cohort study reported no significant associations (Ayano et al., 2022). However, that study had methodological limitations, including a relatively small sample, assessment of academic outcomes at a single time point and the absence of matching, which may have reduced its ability to detect associations.

In this study, we found little or no evidence of an association between perinatal depression and numeracy outcomes. While no previous study has examined the effect of antenatal depression specifically, our findings contrast with earlier studies that reported associations between postnatal depression and children’s numeracy performance (Ahun et al., 2022; Pearson et al., 2016). For example, a cohort study from the United Kingdom reported that adolescents whose mothers experienced postnatal depressive symptoms were 1.5 times more likely to fail mathematics compared to their peers whose mothers did not (Pearson et al., 2016), and a Canadian cohort study similarly reported a negative association between maternal postnatal depressive symptoms and children’s mathematics scores (Ahun et al., 2022). Several methodological differences may account for these discrepancies. None of the existing studies applied matching techniques, increasing the likelihood of residual confounding. Moreover, earlier studies frequently relied on self-reported screening tools such as the Edinburgh Postnatal Depression Scale (EPDS) or the Center for Epidemiologic Studies Depression Scale (CES-D), which are useful for identifying symptoms but lack the diagnostic precision of clinical classifications. Inadequate adjustment for key confounders and reliance on academic outcomes measured at a single time point further limit the comparability of prior findings. It is also important to note that these studies assessed depressive symptoms over extended postpartum periods – up to 5 years – potentially capturing exposures more proximal to academic assessments. By contrast, our study focused specifically on clinically diagnosed postnatal depressive disorders within the first year postpartum, identified using ICD-10-AM codes.

### Potential mechanisms

The mechanisms through which maternal perinatal depressive disorders may affect children’s academic outcomes are multifaceted and interconnected. One explanation is that depression often occurs within families (Sullivan et al., 2000), whereby children of mothers who experience depression have a higher likelihood of developing depression or other mental health conditions than their unexposed peers (Dadi et al., 2020; Tusa et al., 2024, 2025a, 2025b, 2025c). These conditions may negatively affect school performance (Dalsgaard et al., 2020; Wickersham et al., 2021) by impairing attention, endurance, time management, memory and problem-solving skills.

Another pathway involves disruptions to foetal brain development (Cattarinussi et al., 2021). Maternal antenatal depression may alter the intrauterine environment, increasing the risk of neurocognitive impairments in offspring. These impairments may affect executive functioning, processing speed, memory and verbal learning, core skills for academic success (Robinson et al., 2019; Severo et al., 2023). Epigenetic modifications in brain regions involved in learning and memory have also been proposed as mechanisms contributing to later cognitive difficulties (Nemoda and Szyf, 2017). Furthermore, maternal antenatal depression has been associated with adverse birth outcomes such as preterm birth and low birth weight, both of which are linked to increased risks of cognitive and educational difficulties (Dadi et al., 2020; Eves et al., 2021).

Maternal postnatal depression may also interfere with the maturation of functional brain networks underpinning behavioural and cognitive development (Morgan et al., 2021). Neuroimaging research has shown that higher levels of maternal postnatal depression are associated with reduced alpha-range brain connectivity in offspring, which may relate to lower cognitive functioning (Şipoş et al., 2025). Postnatal depression may also affect mother–infant bonding (Sasayama et al., 2025), contributing to behavioural difficulties in children, which can compromise academic performance by affecting motivation, emotional regulation and concentration (McLeod et al., 2012).

Maternal depression also frequently co-occurs with socioeconomic disadvantage (Szurek-Cabanas et al., 2024). Lower socioeconomic status is strongly associated with poorer academic outcomes, partly due to reduced access to high-quality education and fewer opportunities for tertiary study (Vadivel et al., 2023). However, as these factors were accounted for in the present analysis, they are unlikely to fully explain the associations observed.

### Strengths and limitations of the study

This study has several notable strengths. First, the study utilised a large population-based sample, enhancing both statistical power and generalisability. Second, it captured variation in academic performance over time by assessing five scholastic domains at three time points across 5 years of schooling. Third, maternal depressive disorders and key confounders, including other mental health and substance use disorders, were identified using the standardised ICD-10-AM diagnostic system, ensuring diagnostic consistency. Finally, by accounting for key confounders and applying covariate matching, the study minimised bias and residual confounding, increasing confidence that the observed associations reflect true relationships.

Several limitations should be considered when interpreting these findings. First, the exposure definition was restricted to clinically diagnosed depressive disorders occurring during the perinatal period. Some mothers may have experienced persistent or recurrent depression beyond this window. Consequently, the study captures associations related specifically to perinatal depressive disorder as defined within the first year postpartum but does not account for long-term maternal mental health trajectories that may also influence children’s developmental and academic outcomes. Second, maternal and paternal depression commonly co-occur during the perinatal period (Thiel et al., 2020), and evidence suggests that paternal depression is also associated with adverse educational outcomes in children (Shen et al., 2016). Yet, this study did not account for paternal mental health, which may have influenced the observed associations. Third, maternal anxiety and related psychiatric comorbidities were included as covariates to better isolate associations specific to depressive disorders. However, given the substantial symptom overlap between anxiety and depression during the perinatal period, this adjustment may have accounted for shared variance between these conditions. Accordingly, the reported estimates reflect associations specific to depressive disorders independent of measured comorbid anxiety and may therefore represent conservative estimates. Fourth, ascertainment of perinatal depressive disorders was based on hospital admission records, thereby identifying clinically recognised cases requiring hospital-based care. Mothers treated exclusively in primary care or community settings were not captured. As a result, the findings likely reflect associations for more severe or hospital-treated cases and may not be generalisable to milder or subclinical presentations. Finally, the use of historical birth cohorts (2003–2005) may limit the direct generalisability of findings to current clinical practice. Although these cohorts were selected to enable sufficient follow-up for later NAPLAN outcomes, in Grades 5 and 7, perinatal mental health screening practices and service delivery models may have evolved over time. Therefore, caution is required when generalising these results to contemporary practice.

## Conclusion

This large population-based study observed that maternal perinatal depressive disorders were associated with an increased risk of poorer academic performance in children, particularly across literacy domains, including reading, spelling, writing and grammar, whereas associations with numeracy were less evident. The pattern of associations was more consistent for antenatal depression than for postnatal depression. The results contribute population-level evidence that exposure to clinically recognised perinatal depressive disorders may have implications extending into children’s school years. In addition to supporting early identification and management of maternal depression, the findings underscore the importance of coordinated developmental and educational monitoring for children exposed to perinatal depression, with particular attention to literacy-related outcomes.

## Supplemental Material

sj-docx-1-anp-10.1177_00048674261457299 – Supplemental material for Academic performance in children of mothers with perinatal depressive disorder Supplemental material, sj-docx-1-anp-10.1177_00048674261457299 for Academic performance in children of mothers with perinatal depressive disorder by Biruk Shalmeno Tusa, Rosa Alati, Getinet Ayano and Berihun Dachew in Australian & New Zealand Journal of Psychiatry

sj-docx-2-anp-10.1177_00048674261457299 – Supplemental material for Academic performance in children of mothers with perinatal depressive disorder Supplemental material, sj-docx-2-anp-10.1177_00048674261457299 for Academic performance in children of mothers with perinatal depressive disorder by Biruk Shalmeno Tusa, Rosa Alati, Getinet Ayano and Berihun Dachew in Australian & New Zealand Journal of Psychiatry

sj-docx-3-anp-10.1177_00048674261457299 – Supplemental material for Academic performance in children of mothers with perinatal depressive disorder Supplemental material, sj-docx-3-anp-10.1177_00048674261457299 for Academic performance in children of mothers with perinatal depressive disorder by Biruk Shalmeno Tusa, Rosa Alati, Getinet Ayano and Berihun Dachew in Australian & New Zealand Journal of Psychiatry
