# Supplementary material for: Academic performance in children of mothers with perinatal depressive disorder
Source: Aust N Z J Psychiatry. 2026 Jun 23;60(9):870–81. doi: 10.1177/00048674261457299 (PMC13522424; doi:10.1177/00048674261457299)
Supplement: Supplementary file 1 [file sj-docx-1-anp-10.1177_00048674261457299.docx]

Figure S1: The distribution of propensity scores in the treated and control groups (perinatal depressive disorders)

Figure S2**:** The standardised mean differences for each covariate pre- and post-matching (perinatal depressive disorders)

Figure S3 The distribution of propensity scores in the treated and control groups (antenatal depressive disorders)

Figure S4: The standardised mean differences for each covariate pre- and post-matching (antenatal depressive disorders)

Figure S5: The distribution of propensity scores in the treated and control groups (postnatal depressive disorders)

Figure S6: The standardised mean differences for each covariate pre- and post-matching (postnatal depressive disorders
