# Supplementary material for: Academic performance in children of mothers with perinatal depressive disorder
Source: Aust N Z J Psychiatry. 2026 Jun 23;60(9):870–81. doi: 10.1177/00048674261457299 (PMC13522424; doi:10.1177/00048674261457299)
Supplement: Supplementary file 3 [file sj-docx-3-anp-10.1177_00048674261457299.docx]

**Table S2: Univariable and multivariable analysis for the association between offspring educational outcomes and maternal depressive disorder at each year level**

| **Outcome variables** | **Antenatal depressive disorder** | | | | **Postnatal depressive disorder** | | | | **Perinatal depressive disorder** | | | |
| --- | --- | --- | --- | --- | --- | --- | --- | --- | --- | --- | --- | --- |
|  | **Unadjusted OR**  **(95% CI)** | **P-value** | **Adjusted OR^#^**  **(95% CI)** | **P-value** | **Unadjusted OR**  **(95% CI)** | **P-value** | **Adjusted OR^#^***  **(95% CI)** | **P-value** | **Unadjusted OR**  **(95% CI)** | **P-value** | **Adjusted OR^#^**  **(95% CI)** | **P-value** |
| Year 3 | | | | | | | | | | | | |
| Reading domain | | | | | | | | | | | | |
| Unmatched | 2.22 (1.78, 2.77) | <0.01 | 1.47 (1.17, 1.86) | <0.01 | 2.50 (1.88, 3.34) | <0.01 | 1.76 (1.28, 2.41) | <0.01 | 2.34 (1.96, 2.81) | <0.01 | 1.68 (1.38, 2.03) | <0.01 |
| Matched | 1.50 (1.16, 1.93) ^¥^ | <0.01 | 1.49 (1.15, 1.95) ^¥^ | <0.01 | 1.83 (1.29, 2.60) ^$^ | <0.01 | 1.90 (1.31, 2.76) ^$^ | <0.01 | 1.55 (1.25, 1.91) ^$^ | <0.01 | 1.60 (1.29, 1.99) ^$^ | <0.01 |
| Spelling domain | | | | | | | | | | | | |
| Unmatched | 2.52 (2.04, 3.11) | <0.01 | 1.53 (1.22, 1.92) | <0.01 | 2.46 (1.84, 3.31) | <0.01 | 1.51 (1.10, 2.09) | 0.01 | 2.48 (2.07, 2.96) | <0.01 | 1.59 (1.31, 1.93) | <0.01 |
| Matched | 1.45 (1.14, 1.85) ^¥^ | <0.01 | 1.46 (1.13, 1.88) ^¥^ | <0.01 | 1.48 (1.05, 2.08) ^$^ | 0.03 | 1.40 (0.97, 2.02) ^$^ | 0.07 | 1.51 (1.23, 1.85) ^$^ | <0.01 | 1.54 (1.24, 1.91) ^$^ | <0.01 |
| Grammar domain | | | | | | | | | | | | |
| Unmatched | 1.71 (1.35, 2.16) | <0.01 | 1.12 (0.87, 1.43) | 0.37 | 1.81 (1.32, 2.48) | <0.01 | 1.23 (0.87, 1.73) | 0.24 | 1.77 (1.46, 2.14) | <0.01 | 1.20 (0.98, 1.47) | 0.09 |
| Matched | 1.19 (0.91, 1.56) ^¥^ | 0.20 | 1.18 (0.89, 1.55) ^¥^ | 0.25 | 1.49 (1.03, 2.17) ^$^ | 0.04 | 1.47 (0.98, 2.20) ^$^ | 0.06 | 1.39 (1.11, 1.73) ^$^ | <0.01 | 1.39 (1.10, 1.75) ^$^ | 0.01 |
| Writing domain | | | | | | | | | | | | |
| Unmatched | 2.44 (1.84, 3.24) | <0.01 | 1.58 (1.17, 2.12) | <0.01 | 1.82 (1.17, 2.84) | <0.01 | 1.09 (0.68, 1.75) | 0.73 | 2.21 (1.73, 2.82) | <0.01 | 1.44 (1.11, 1.88) | 0.01 |
| Matched | 1.47 (1.06, 2.04) ^¥^ | 0.02 | 1.51 (1.08, 2.11) ^¥^ | 0.02 | 1.02 (0.61, 1.71) | 0.93 ^$^ | 0.93 (0.54, 1.59) ^$^ | 0.78 | 1.47 (1.10, 1.96) ^$^ | 0.01 | 1.51 (1.12, 2.02) ^$^ | 0.01 |
| Numeracy domain | | | | | | | | | | | | |
| Unmatched | 1.52 (1.15, 2.00) | <0.01 | 0.99 (0.74, 1.32) | 0.94 | 1.55 (1.06, 2.26) | 0.02 | 1.08 (0.72, 1.62) | 0.70 | 1.50 (1.19, 1.89) | <0.01 | 1.01 (0.79, 1.29) | 0.95 |
| Matched | 0.97 (0.72, 1.32) ^¥^ | 0.86 | 0.97 (0.71, 1.33) ^¥^ | 0.83 | 1.12 (0.73, 1.73) ^$^ | 0.59 | 1.11 (0.70, 1.74) ^$^ | 0.66 | 0.99 (0.76, 1.28) ^$^ | 0.93 | 0.97 (0.75, 1.27) ^$^ | 0.83 |
| Any domain | | | | | | | | | | | | |
| Unmatched | 1.96 (1.69, 2.27) | <0.01 | 1.30 (1.11, 1.53) | <0.01 | 1.99 (1.62, 2.44) | <0.01 | 1.37 (1.09, 1.72) | 0.01 | 1.97 (1.74, 2.22) | <0.01 | 1.37 (1.19, 1.57) | <0.01 |
| Matched | 1.32 (1.11, 1.57) ^¥^ | <0.01 | 1.33 (1.11, 1.59) ^¥^ | <0.01 | 1.46 (1.15, 1.85) ^$^ | <0.01 | 1.43 (1.10, 1.86) ^$^ | 0.01 | 1.38 (1.19, 1.59) ^$^ | <0.01 | 1.41 (1.21, 1.64) ^$^ | <0.01 |
| Year 5 | | | | | | | | | | | | |
| Reading domain | | | | | | | | | | | | |
| Unmatched | 1.81 (1.47, 2.23) | <0.01 | 1.21 (0.97, 1.52) | 0.10 | 1.56 (1.15, 2.13) | 0.01 | 1.03 (0.74, 1.44) | 0.09 | 1.76 (1.47, 2.10) | <0.01 | 1.20 (0.99, 1.45) | 0.06 |
| Matched | 1.50 (1.16, 1.93) ^¥^ | <0.01 | 1.49 (1.15, 1.95) ^¥^ | <0.01 | 1.05 (0.73, 1.50) ^$^ | 0.79 | 1.02 (0.69, 1.49) ^$^ | 0.94 | 1.22 (0.99, 1.49) ^$^ | 0.06 | 1.23 (1.00, 1.52) ^$^ | 0.05 |
| Spelling domain | | | | | | | | | | | | |
| Unmatched | 2.29 (1.89, 2.78) | <0.01 | 1.45 (1.18, 1.78) | <0.01 | 2.03 (1.53, 2.68) | <0.01 | 1.27 (0.93, 1.72) | 0.13 | 2.12 (1.80, 2.50) | <0.01 | 1.38 (1.16, 1.66) | <0.01 |
| Matched | 1.45 (1.14, 1.85) ^¥^ | <0.01 | 1.46 (1.13, 1.88) ^¥^ | <0.01 | 1.25 (0.89, 1.75) ^$^ | 0.20 | 1.17 (0.82, 1.68) ^$^ | 0.38 | 1.34 (1.11, 1.63) ^$^ | <0.01 | 1.37 (1.12, 1.67) ^$^ | <0.01 |
| Grammar domain | | | | | | | | | | | | |
| Unmatched | 1.74 (1.42, 2.13) | <0.01 | 1.14 (0.92, 1.41) | 0.23 | 1.69 (1.27, 2.24) | <0.01 | 1.13 (0.83, 1.54) | 0.45 | 1.68 (1.41, 2.00) | <0.01 | 1.13 (0.94, 1.36) | 0.21 |
| Matched | 1.19 (0.91, 1.56) ^¥^ | 0.20 | 1.18 (0.89, 1.55) ^¥^ | 0.25 | 1.20 (0.87, 1.67) ^$^ | 0.27 | 1.18 (0.83, 1.67) ^$^ | 0.35 | 1.21 (0.99, 1.47) ^$^ | 0.06 | 1.21 (0.99, 1.48) ^$^ | 0.06 |
| Writing domain | | | | | | | | | | | | |
| Unmatched | 2.15 (1.80, 2.56) | <0.01 | 1.32 (1.09, 1.60) | 0.01 | 2.02 (1.57, 2.60) | <0.01 | 1.21 (0.92, 1.60) | 0.18 | 2.09 (1.80, 2.42) | <0.01 | 1.31 (1.11, 1.55) | <0.01 |
| Matched | 1.47 (1.06, 2.04) ^¥^ | 0.02 | 1.51 (1.08, 2.11) ^¥^ | 0.02 | 1.37 (1.02, 1.83) ^$^ | 0.04 | 1.32 (0.96, 1.82) ^$^ | 0.09 | 1.31 (1.10, 1.55) ^$^ | <0.01 | 1.30 (1.08, 1.56) ^$^ | 0.01 |
| Numeracy domain | | | | | | | | | | | | |
| Unmatched | 1.43 (1.11, 1.83) | <0.01 | 0.96 (0.74, 1.26) | 0.76 | 1.53 (1.09, 2.14) | 0.01 | 1.07 (0.74, 1.26) | 0.71 | 1.47 (1.20, 1.81) | <0.01 | 1.01 (0.81, 1.26) | 0.93 |
| Matched | 0.97 (0.72, 1.32) ^¥^ | 0.86 | 0.97 (0.71, 1.33) ^¥^ | 0.83 | 1.00 (0.68, 1.49) ^$^ | 0.97 | 0.98 (0.65, 1.48) ^$^ | 0.93 | 1.02 (0.81, 1.28) ^$^ | 0.88 | 1.01 (0.80, 1.28) ^$^ | 0.92 |
| Any domain | | | | | | | | | | | | |
| Unmatched | 1.97 (1.73, 2.24) | <0.01 | 1.34 (1.16, 1.54) | <0.01 | 1.67 (1.38, 2.02) | <0.01 | 1.10 (0.89, 1.36) | 0.38 | 1.83 (1.64, 2.05) | <0.01 | 1.27 (1.12, 1.43) | <0.01 |
| Matched | 1.32 (1.11, 1.57) ^¥^ | <0.01 | 1.33 (1.11, 1.59) ^¥^ | <0.01 | 1.09 (0.88, 1.36) ^$^ | 0.43 | 1.03 (0.81, 1.31) ^$^ | 0.82 | 1.24 (1.09, 1.41) ^$^ | <0.01 | 1.26 (1.10, 1.44) ^$^ | <0.01 |
| Year 7 |  |  |  |  |  |  |  |  |  |  |  |  |
| Reading domain | | | | | | | | | | | | |
| Unmatched | 1.89 (1.54, 2.34) | <0.01 | 1.20 (0.96, 1.50) | 0.11 | 1.92 (1.44, 2.56) | <0.01 | 1.19 (0.87, 1.63) | 0.27 | 1.87 (1.57, 2.23) | <0.01 | 1.20 (1.00, 1.46) | 0.06 |
| Matched | 1.13 (0.89, 1.43) ^¥^ | 0.32 | 1.14 (0.89, 1.46) ^¥^ | 0.30 | 1.22 (0.87, 1.70) ^$^ | 0.25 | 1.25 (0.88, 1.79) ^$^ | 0.22 | 1.11 (0.91, 1.35) ^$^ | 0.32 | 1.11 (0.91, 1.37) ^$^ | 0.31 |
| Spelling domain | | | | | | | | | | | | |
| Unmatched | 1.99 (1.64, 2.42) | <0.01 | 1.27 (1.03, 1.56) | 0.02 | 1.96 (1.50, 2.57) | <0.01 | 1.28 (0.96, 1.72) | 0.10 | 1.88 (1.60, 2.22) | <0.01 | 1.24 (1.04, 1.49) | 0.02 |
| Matched | 1.13 (0.91, 1.41) ^¥^ | 0.27 | 1.14 (0.90, 1.43) ^¥^ | 0.28 | 1.10 (0.80, 1.51) ^$^ | 0.55 | 1.07 (0.77, 1.50) ^$^ | 0.68 | 1.19 (0.98, 1.43) ^$^ | 0.07 | 1.22 (1.00, 1.48) ^$^ | 0.05 |
| Grammar domain | | | | | | | | | | | | |
| Unmatched | 1.75 (1.46, 2.10) | <0.01 | 1.15 (0.95, 1.40) | 0.15 | 1.73 (1.34, 2.22) | <0.01 | 1.15 (0.88, 1.52) | 0.31 | 1.71 (1.47, 1.99) | <0.01 | 1.15 (0.98, 1.36) | 0.09 |
| Matched | 1.06 (0.86, 1.30) ^¥^ | 0.59 | 1.05 (0.85, 1.30) ^¥^ | 0.64 | 1.14 (0.85, 1.53) ^$^ | 0.40 | 1.14 (0.83, 1.56) ^$^ | 0.43 | 1.09 (0.91, 1.29) ^$^ | 0.35 | 1.09 (0.91, 1.31) ^$^ | 0.33 |
| Writing domain | | | | | | | | | | | | |
| Unmatched | 2.08 (1.79, 2.42) | <0.01 | 1.32 (1.12, 1.56) | <0.01 | 1.93 (1.56, 2.39) | <0.01 | 1.20 (0.94, 1.53) | 0.14 | 2.02 (1.78, 2.30) | <0.01 | 1.32 (1.15, 1.53) | <0.01 |
| Matched | 1.25 (1.05, 1.48) ^¥^ | 0.01 | 1.29 (1.07, 1.55) ^¥^ | 0.01 | 1.25 (0.98, 1.60) ^$^ | 0.07 | 1.32 (1.01, 1.73) ^$^ | 0.05 | 1.24 (1.07, 1.43) ^$^ | <0.01 | 1.27 (1.09, 1.49) ^$^ | <0.01 |
| Numeracy domain | | | | | | | | | | | | |
| Unmatched | 2.13 (1.63, 2.79) | <0.01 | 1.26 (0.95, 1.67) | 0.11 | 2.54 (1.81, 3.58) | <0.01 | 1.51 (1.04, 2.20) | 0.03 | 2.20 (1.77, 2.74) | <0.01 | 1.35 (1.06, 1.71) | 0.01 |
| Matched | 1.36 (1.00, 1.84) ^¥^ | 0.05 | 1.32 (0.96, 1.82) ^¥^ | 0.08 | 1.39 (0.93, 2.08) ^$^ | 0.11 | 1.42 (0.93, 2.18) ^$^ | 0.11 | 1.29 (1.00, 1.67) ^$^ | 0.05 | 1.31 (1.00, 1.70) ^$^ | 0.05 |
| Any domain | | | | | | | | | | | | |
| Unmatched | 1.99 (1.76, 2.25) | <0.01 | 1.32 (1.15, 1.52) | <0.01 | 1.75 (1.46, 2.09) | <0.01 | 1.14 (0.93, 1.39) | 0.22 | 1.88 (1.69, 2.09) | <0.01 | 1.27 (1.13, 1.43) | <0.01 |
| Matched | 1.19 (1.04, 1.37) ^¥^ | 0.01 | 1.21 (1.04, 1.41) ^¥^ | 0.01 | 1.14 (0.93, 1.40) ^$^ | 0.20 | 1.15 (0.91, 1.44) ^$^ | 0.23 | 1.18 (1.05, 1.33) ^$^ | 0.01 | 1.21 (1.06, 1.37) ^$^ | <0.01 |
| *^#^ Models adjusted for maternal age, socio-economic indicators, language background other than English, sex of the baby, birth order, plurality of birth parity, presentation, labour onset, mode of delivery, preterm, low birth weight, low Apgar score, admitted to neonatal intensive care unit, admitted to a special care nursery, maternal antenatal infection, maternal antenatal smoking, maternal antenatal anaemia, chronic hypertension, pregnancy-induced hypertension, pre-existing diabetes, gestational diabetes, perinatal anxiety disorder, perinatal bipolar disorder, perinatal schizophrenia disorder, perinatal alcohol use disorder, and perinatal substance use disorder.*  *^*^Further adjusted for antenatal depressive disorder*  *$: The sample matched for all variables in models*  *¥: The sample matched for all variables in the models except labour onset, mode of delivery, presentation, preterm, low birth weight, low Apgar score, admitted to the neonatal intensive care unit, and admitted to a special care nursery* | | | | | | | | | | | | |
